# Effectiveness and safety of acupuncture in post-stroke depression (PSD): Protocol for a Bayesian analysis

**DOI:** 10.1097/MD.0000000000018969

**Published:** 2020-03-20

**Authors:** Baishu Chen, Minhong Zhao, Bin Chen, Zhaojun Yang, Xiaojiang Yu, Xueying Lin, Chun Fan

**Affiliations:** a Baoan Hospital of Traditional Chinese Medicine in Shenzhen, Shenzhen, Guangdong; b Clinical Medical College of Acup-moxibustion & Rehabilitation, Guangzhou University of Chinese Medicine; c Guangzhou University of Chinese Medicine; d Department of Rehabilitation Center, First Affiliated Hospital of Guangzhou University of Chinese Medicine, Guangzhou, China.

**Keywords:** acupuncture, Bayesian analysis, post-stroke depression

## Abstract

**Background:**

Post-stroke depression (PSD) is the most common emotional problem after stroke. It can lower the quality of life and increase the recurrence and mortality. Pharmacological agents have been shown to treat PSD. However, the benefits of pharmacotherapy are debatable and the side-effects are significant. More and more clinical trials suggest that acupuncture plays an important role in patients with PSD. The primary purposes of the study are to conduct a Bayesian analysis of randomized trials to determine the effect of acupuncture and investigate the effect of several acupuncture therapies on PSD.

**Methods:**

We will retrieve articles from 5 English databases [PubMed, Web of Science, EMBASE, the Cochrane Central Register of Controlled Trials, and WHO International Clinical Trials Registry (TCTRP)] and 4 Chinese databases [Chinese National Knowledge Infrastructure (CNKI), Chinese VIP Information, Wanfang Database, and Chinese Biomedical Literature Database (CBM)]. The publication period will be from inception to January 2019. All randomized controlled trials that evaluate the safety and efficacy of acupuncture on PSD will be included. The primary outcomes will be the change in the degree of depression as measured by the Hamilton Depression Scale and Beck Depression Inventory. Two reviewers will separately extract the data and assess the risk of bias by using the Cochrane Collaboration's risk of bias tool. Bayesian analysis will be conducted to pool the effects of several acupunctures. The ranking probabilities for several acupunctures (simple acupuncture, fire needle, warm acupuncture, auriculo-acupuncture, or electroacupuncture) will be estimated by the surface under the cumulative ranking curve.

**Result:**

This study will provide reliable evidence for acupuncture on PSD.

**Conclusion:**

The results of this review will introduce a safe and effective treatment and provide reliable evidence to evaluate the effectiveness of several acupuncture therapies on PSD.

Trial registration number: CRD42019132725

## 1 Introduction

### 1.1 Description of the condition

Post-stroke depression (PSD) is the most common emotional problem after stroke.^[1]^ Mostly, survivors after stroke experience considerable long-term impairments in sensory and motor coordination, cognition, and behavior,^[2]^ which often limit normal daily life activities.^[3]^ Previous studies showed over 30% of the patients experienced severe emotional complaints.^[4–5]^ The underlying mechanism of PSD is still unclear. Recent articles revealed the possible etiologies that included psychosocial factors and biological factors, such as the grief of disability and proinflammatory cytokine release.^[6–7]^

PSD is commonly treated with pharmacological agents, such as citalopram, escitalopram, nortriptyline, milnacipran, mirtazapine, piracetam, and fluoxetine.^[8]^ However, the benefits of pharmacotherapy are debatable^[9]^ and the side-effects including blurry vision, hypotension, and severe insomnia are reported.^[10]^

PSD can lower the quality of life, slow down the health recovery, and increase the recurrence and mortality of stroke.^[11]^ Researches showed up to 60% of patients respond inadequately to pharmacological antidepressant treatment^[12]^ and 30% could not ensure adherence to long-term treatment because of the side-effects.^[13]^ It is necessary to develop a safe and effective treatment plan for PSD. The research about assessing the effectiveness of TCM therapies is increasing.

### 1.2 Description of the intervention

**Figure 1:**
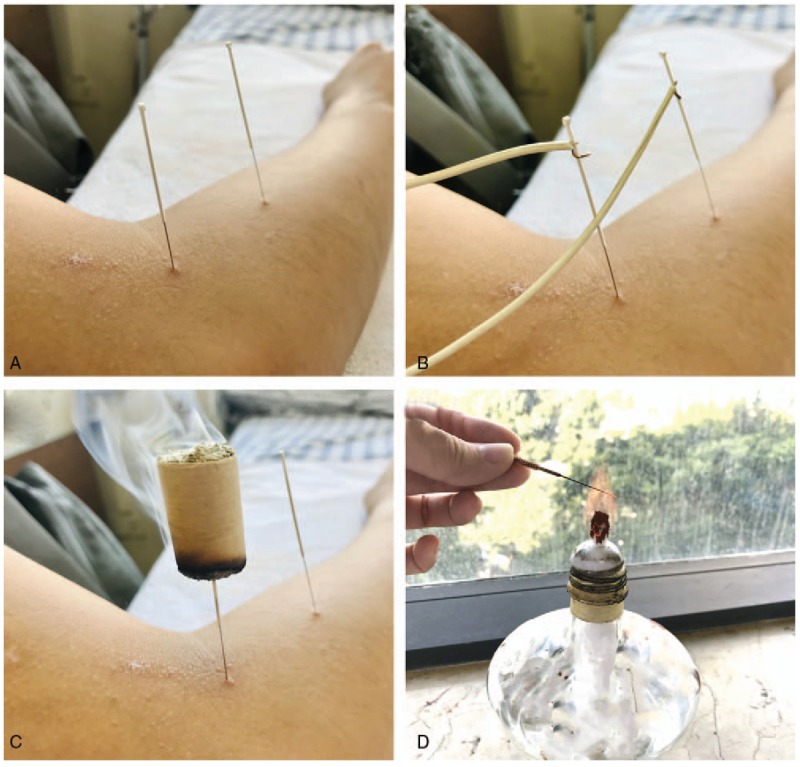
Several forms of acupunctures.

Acupuncture, which uses needles to puncture into acupoints, is one of the most popular traditional Chinese medicine (TCM). Acupuncture includes several forms, such as simple acupuncture, fire needle, warm acupuncture, auriculo-acupuncture, and electroacupuncture (Fig. 1). It can achieve the goal of treating diseases by enhancing immunity, regulating blood circulation,^[14]^ modulating relevant molecules,^[15]^ and activating related brain regions.^[16]^ Some researches found no convincing evidence that acupuncture improved lifestyle risk factors of stroke.^[17]^ However, other studies showed acupuncture was efficient in stroke patients.^[18]^ In addition, compared to pharmacological agents, acupuncture has the advantages of cheap, safe, reliable, and easy to use without side effects. But which form of acupuncture has better clinical efficacy still remained unclear.

**Figure 2:**
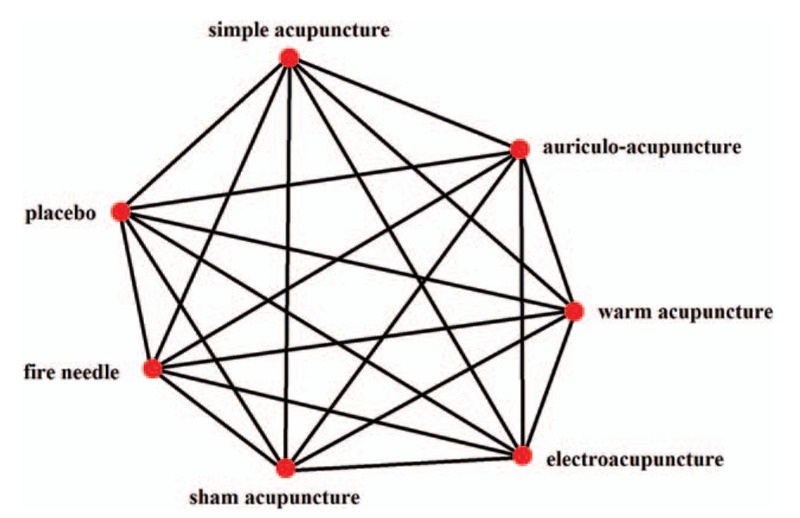
Network plot of possible direct comparisons between the interventions.

In this study, we use Bayesian analysis to summarize clinical trials and investigate the effect of several acupuncture therapies on PSD (Fig. 2).

## 2 Method

### 2.1 Study registration

The protocol, whose registration number is CRD42019132725, has been registered on the International Prospective Register of Systematic Reviews (PROSPERO). It is based on the Preferred Reporting Items for Systematic Reviews and Meta-Analyses Protocols (PRISMA-P) statement guidelines, and the checklist will be upload as an attachment.

### 2.2 Eligibility criteria

#### 2.2.1 Study type

We will include all randomized controlled trials that evaluate the safety and efficacy of acupuncture on PSD (Fig. 2). Retrospective study, case report, review, and studies in which stroke and depressive disorder are not formally diagnosed will be excluded.

#### 2.2.2 Participants

We will include studies on patients who have a recent or past history of ischemic or hemorrhagic stroke and have been diagnosed as depressive disorder, based on *All Kinds of Cerebrovascular Disease Diagnosis Points*^[19]^ and *Diagnostic and Statistical Manual of Mental Disorders*.^[20]^ Enrolled participants will be medically stable. What is more, they will be able to give informed consent and follow multiple-staged commands. There is no restriction on age, gender, ethnicity, and profession.

#### 2.2.3 Intervention

Intervention group patients will receive one form of acupuncture, such as simple acupuncture, fire needle, warm acupuncture, auriculo-acupuncture, or electroacupuncture (Fig. 1). There is no restriction on duration and frequency. Acupuncture combined with other therapies will be excluded if the efficacy of acupuncture cannot be clarified. Control group patients will receive the treatment of placebo or sham acupuncture.

#### 2.2.4 Outcome measure

##### 2.2.4.1 Primary outcome

The primary outcome is the change in the degree of depression as measured by assessment tools such as the Hamilton Depression Scale^[21]^ and Beck Depression Inventory.^[22]^

##### 2.2.4.2 Secondary outcome

- Changes in activities of daily living measured by validated assessment tools such as the Barthel Index^[23]^
- Quality of life measured by validated assessment tools such as The Stroke Specific Quality of Life Scale^[24]^ and the 36-Item Short Form Health Survey^[25]^
- Syndrome according to standards for assessing TCM
- Adverse events

### 2.3 Search methods

We will comprehensively search articles from 5 English databases [PubMed, Web of Science, EMBASE, the Cochrane Central Register of Controlled Trials, and WHO International Clinical Trials Registry (TCTRP)] and 4 Chinese databases [Chinese National Knowledge Infrastructure (CNKI), Chinese VIP Information, Wanfang Database, and Chinese Biomedical Literature Database (CBM)]. The publication period will be from inception to January 2019. The search items will include “electro-acupuncture” OR “warm needle acupuncture” OR “fire needle” OR “auriculo-acupuncture” OR “acupuncture” AND “Stroke” OR “Cerebrovascular disease” OR “Vascular Diseases” OR “Acute Cerebrovascular Accident” OR “Brain Vascular Accident” OR “Acute Stroke” AND “depressive disorder” OR “depression” OR “depressive.” The detailed search strategies in PubMed are shown in Table 1 and Chinese translations of these items will be used for the Chinese databases. Moreover, relevant medical journals and magazines which are not included in the electronic databases will be searched too.

**Table 1:**
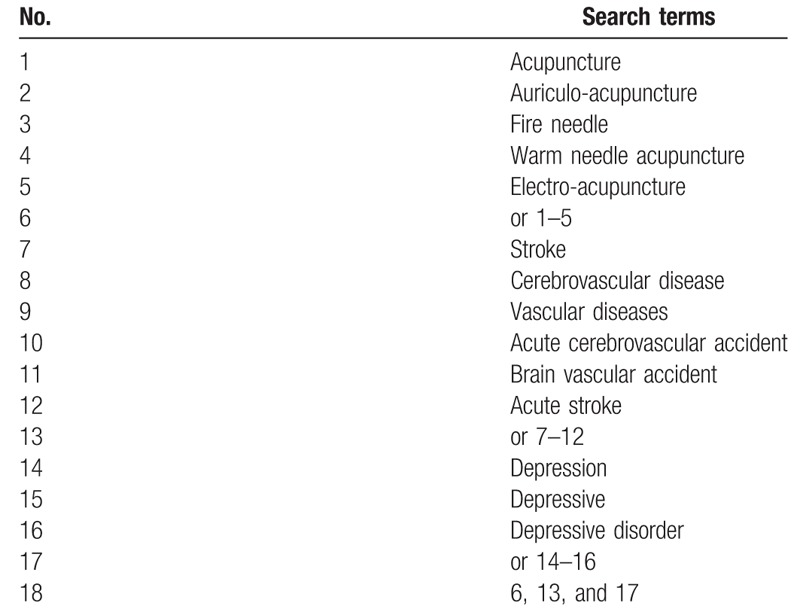
Search strategy utilized for the PubMed database.

### 2.4 Data extraction

Two reviewers will separately screen titles and abstracts of studies based on eligible criteria in EndNote X7. After elementary screening, full texts of eligible studies will be reviewed. In this process, the following data will be extracted:

1. general information (e.g., author list and publication year);
2. participants (e.g., age, gender, sample size, and weight);
3. details about intervention (e.g., acupuncture parameters, acupuncture points, and duration of study);
4. study design, randomization, blinding, and allocation concealment;
5. outcome, including adverse effects.

**Figure 3:**
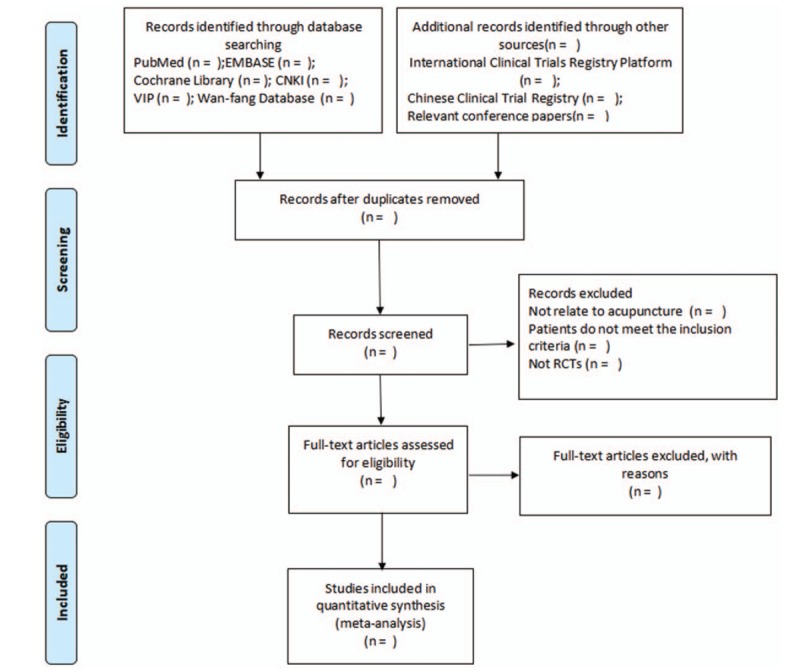
Flow diagram of the study selection process.

A third reviewer will discuss and solve the discrepancies about inclusion in the net-work analysis. The study selection will be showed by a PRISMA flow chart (http://www.prisma-statement.org) (Fig. 3).

### 2.5 Quality assessment

Two independent researchers will assess the included studies by using the Cochrane Collaboration's risk of bias tool. The following aspects will be focused on: random sequence generation, allocation concealment, blinding of participants and personnel, blinding of outcome assessments, incomplete outcome data, selective reporting. Studies will be categorized into 3 groups: “low risk,” “unclear,” or “high risk.” Discrepancies will be resolved through discussion.

### 2.6 Data analysis

#### 2.6.1 Data synthesis and statistical analysis

Data synthesis and analysis will be performed using Stata V.13.0 software and will be shared among researchers in Dropbox (Dropbox, Inc) folders. Dichotomous data will be measured by odd ratios (OR) and 95% confidence intervals (CI). Meanwhile, the evaluation for the consistency of the closed-loop in the interventions network will not be neglected. Consistency test will be performed to assess the discrepancies between direct and indirect comparisons among several acupuncture interventions. With the tool of WinBUGS 1.4.3 software, the surface under the cumulative ranking curve (SUCRA) will be conducted to sequence the probabilities of the optimal intervention of various treatments. A lager area under the curve will represent a better rank of intervention. To assess reporting bias of the studies included, funnel plots will be developed.

#### 2.6.2 Subgroup analysis

If there is enough clinical data, subgroup analysis will be divided by different course, age, controls, outcome measures, and interventions. The heterogeneity in trials is significant when I^2^ ≥ 50%.

#### 2.6.3 Sensitivity analysis

If the heterogeneity is high, sensitivity analysis will be performed on each indicator to find the source of heterogeneity. Network meta-regression will be conducted to discover inconsistent sources.

#### 2.6.4 Quality of evidence

We will evaluate the quality of included studies by GRADE (Grading of Recommendations, Assessment, Development, and Evaluation) and Grade profiler 3.6 will be used. According to GRADE, there will be 4 levels of evidence: high, medium, low, and very low.

#### 2.6.5 Patient and public involvement

Patients and public will not be involved.

#### 2.6.6 Ethics and dissemination

We do not include individual patient data, so ethical approval is not required. The results will be disseminated through a peer-reviewed publication. The outcomes from this trial will find out the best form of acupuncture in treating PSD.

## 3 Discussion

Depression, which is related to poor functional outcome and high mortality, is common after stroke.^[26]^ Global burden of diseases report describes stroke with depression as the “double burden” of stroke.^[12]^ The most commonly used antidepressant is pharmacological agents which can cause adverse effects. Moreover, the placebo effects of this therapy should not be ignored. Therefore, it is important to explore other effective treatments that may have fewer side effects. Acupuncture is a good choice.

Acupuncture is a traditional Chinese therapeutic intervention which inserts fine metallic needles through the skin at specific sites.^[27]^ Acupuncture also has antidepressant effect.^[28]^ Study has found that acupuncture can mediate depressive emotion by regulating the deoxyribonucleic acid (DNA) methylation and histone modifications of brain-derived neurotrophic factor (BDNF).^[29]^ Further research supported that acupuncture could ameliorate depressive-like behaviors by regulating PKA/CREB signaling pathway.^[30]^ By comparing the expression levels of related indicators before and after treatment, a 4-week electro-acupuncture treatment on rats with behavioral deficits has showed antidepressant effect.^[31]^ The effectiveness of acupuncture combined with antidepressant medication on depression has been proved in a systematic review.^[32]^ However, there has been no relevant network analysis that show the effect of several acupunctures on improving PSD and which form of acupuncture is better on PSD is still unknown. In addition, there is no sufficient evidence to support the widespread use of acupuncture on PSD. Hence, a network-analysis about the effect of several forms of acupuncture on improved PSD is needed. The purpose of this review is to systematically assess the effect of different forms of acupuncture on PSD. We hope to find out the better form of acupuncture that helps improving depression.

To the best of our knowledge, this review will be the first to evaluate the effect of several forms of acupuncture on PSD. We believe that the results of this review will introduce a safe and effective treatment and provide reliable evidence for its application.

## Author Contributions

Baishu Chen and Minhong Zhao contributed equally to this paper and are co-first authors. Chun Fan conceived of the study. Baishu Chen and Minhong Zhao design the systematic review. Bin Chen and Zhaojun Yang revised it. Xiaojiang Yu and Xueying Lin developed the search strategies and conducted data collection. All authors have approved the final manuscript.

**Conceptualization:** Chun Fan

**Formal analysis:** Baishu Chen, Minhong Zhao

**Methodology:** Xiaojiang Yu, Xueying Lin

**Writing – original draft:** Bin Chen, Zhaojun Yang

**Writing – review & editing:** Minhong Zhao, Chun Fan
